# Proteomic analysis-based discovery of a novel biomarker that differentiates intestinal Behçet’s disease from Crohn’s disease

**DOI:** 10.1038/s41598-021-90250-2

**Published:** 2021-05-26

**Authors:** Jihye Park, Daeun Jeong, Youn Wook Chung, Seunghan Han, Da Hye Kim, Jongwook Yu, Jae Hee Cheon, Ji-Hwan Ryu

**Affiliations:** 1 Department of Internal Medicine, Yonsei University College of Medicine, 50-1 Yonsei-ro, Seodaemun-gu, Seoul, 03722 Korea; 2 Institute of Gastroenterology, Yonsei University College of Medicine, Seoul, Korea; 3 Severance Biomedical Science Institute, Yonsei University College of Medicine, 50-1 Yonsei-ro, Seodaemun-gu, Seoul, 03722 Korea; 4 Brain Korea 21 PLUS Project for Medical Science, Yonsei University College of Medicine, Seoul, Korea; 5 Airway Mucus Institute, Yonsei University College of Medicine, Seoul, 03722 Korea

**Keywords:** Biomarkers, Diagnostic markers

## Abstract

Intestinal Behçet’s disease (BD) and Crohn’s disease (CD) present similar manifestations, but there are no specific diagnostic tests to differentiate them. We used a proteomic approach to discover novel diagnostic biomarkers specific to intestinal BD. Colon mucosa tissue samples were obtained from patients with intestinal BD or CD using colonoscopy-guided biopsy of the affected bowel. Peptides from seven intestinal BD and seven CD patients were extracted and labeled using tandem mass tag (TMT) reagents. The labeled peptides were identified and quantified using liquid chromatography-tandem mass spectrometry (LC–MS/MS). The proteins were further validated using immunohistochemical (IHC) analysis with tissue samples and an ELISA test with serum samples from 20 intestinal BD and 20 CD patients. Using TMT/LC–MS/MS-based proteomic quantification, we identified 39 proteins differentially expressed between intestinal BD and CD. Beta-2 glycoprotein 1 (APOH) and maltase-glucoamylase (MGAM) showed higher intensity in the IHC staining of intestinal BD tissues than in CD tissues. The serum MGAM level was higher in intestinal BD patients. Proteomic analysis revealed that some proteins were differentially expressed in patients with intestinal BD compared with those with CD. Differential MGAM expression in intestinal BD suggests its role as a potential novel diagnostic biomarker.

## Introduction

Behçet’s disease (BD) is an idiopathic, chronic, relapsing, multi-systemic vasculitis characterized by recurrent oral or genital ulcers, arthritis, and ocular, dermal, neurovascular, and gastrointestinal manifestations^1,2^. Intestinal BD is diagnosed when a patient with BD has clinical gastrointestinal symptoms and typical endoscopic findings^3^. It is a very rare disease, but the prevalence of BD is the highest in countries located along the Silk Road stretching from Asia to the Mediterranean countries^4^. Crohn’s disease (CD) is a chronic, relapsing, inflammatory, bowel disease (IBD) that may affect the gastrointestinal tract from the mouth to the anus. Once considered to be a Western disease, the incidence of CD has rapidly increased in East Asian countries, such as Japan, Korea, and Hong Kong, while plateauing in the West^5,6^.

Both intestinal BD and CD often present similar gastrointestinal and extraintestinal manifestations, as well as endoscopic findings. Therefore, it is sometimes difficult to differentiate between these two diseases. Clinically, oral and genital ulcerations are more common in intestinal BD patients, and perianal lesions are more common in CD patients^7^. However, both diseases develop nonspecific, waxing and waning, life-long gastrointestinal symptoms^8^. Moreover, ocular and skin manifestations, and arthropathy, can occur in both diseases^9^. Endoscopically, a typical intestinal BD ulcer is characterized by being single or few in number, round or oval in shape, deep, possessing a sharp margin, being larger than 10 mm, and showing localization to the ileocecal valve^10^. The “atypical” intestinal BD ulcer without the systemic manifestations could be confused with CD. There have been several attempts to develop a diagnostic biomarker to differentiate between the two diseases, but there are practically no available tests because of several limitations.

Proteomic analysis is a promising technology that is a powerful tool for identifying biomarkers to help diagnose and choose personalized treatment. It allows high-throughput study of protein expression with high accuracy, sensitivity, and repeatability, and it enables the identification of molecular mechanisms that are responsible for the development of a specific disease^11,12^. The tandem mass tag (TMT)-liquid chromatography-tandem mass spectrometric (LC–MS/MS) method has been recently developed to identify and quantify proteins. Although several studies have investigated colon mucosal biopsies using gel-based proteomic approaches to identify protein biomarkers that differentiate intestinal BD from IBD^13,14^, no applicable biomarkers are currently available. Since gel-free proteomic approaches have several advantages compared to gel-based approaches, particularly for identifying membrane-bound and/or glycosylated large proteins (e.g., mucins)^15^, we used gel-free approaches to analyze intestinal BD proteomes in comparison with CD proteomes.

Here, we aimed to develop diagnostic biomarkers to differentiate between intestinal BD and CD. We employed a coupled TMT/LC–MS/MS-based method to identify proteins that were differentially expressed between patients with intestinal BD and CD.

## Methods

### Study population and sample collection

In total, 47 patients with intestinal BD and 47 patients with CD were recruited from the IBD Clinic of Severance Hospital, Seoul, Korea. Intestinal BD and CD were diagnosed according to clinical, histological, endoscopic, and radiological criteria^16,17^. Exclusion criteria included indeterminate colitis, ulcerative colitis, intestinal tuberculosis, a history of malignancy, or insufficient available medical records. The intestinal BD ulcer type was defined according to the Korean Inflammatory Bowel Disease study group, and the CD phenotype was defined according to the Montreal Classification^18,19^. The severity of intestinal BD was determined using the Disease Activity Index for Intestinal Behçet’s Disease (DAIBD) score, and CD severity was determined using the Crohn’s Disease Activity Index (CDAI) score^20,21^.

The intestinal mucosa tissue samples were obtained from patients with intestinal BD or CD using colonoscopy-guided biopsy of the affected bowel. The tissue and serum samples were preserved at −80 °C.

Informed consent was obtained from all individuals enrolled in this study. This study was approved by the Institutional Review Board of Yonsei University College of Medicine (IRB No: 2012-0039-030) and was conducted in accordance with the Declaration of Helsinki.

### TMT sample processing and protein quantitation

For proteomic analysis, the colon mucosa tissue samples were lysed and labeled with tandem mass tag (TMT, Thermo Scientific, San Jose, CA, USA) according to the manufacturer’s instructions. The labeled peptide samples were pooled into a new vial and dried using SpeedVac (Thermo Scientific). The following processes, including strong cation exchange fraction and liquid chromatography (LC)-mass spectrometry (MS) and database searching, were performed by Poochon Scientific (Frederick, MD) as described previously^22^. Briefly, TMT-multiplex labeled peptide mixture (100 µg protein/each plex) fractionation was performed using an Agilent AdvanceBio Column and Agilent UHPLC 1290 system (Agilent, Santa Clara, CA). LC/MS/MS analysis was performed using a Thermo Scientific Q-Exactive hybrid Quadrupole-Orbitrap Mass Spectrometer and Thermo Dionex UltiMate 3000 RSLCnano System (Thermo Scientific). Raw MS data files were searched against the human protein sequence databases obtained from the NCBI website using Proteome Discoverer 1.4 software (Thermo Scientific) based on the SEQUEST and percolator algorithms. The false positive discovery rate (FDR) was set at 5%. The resulting Proteome Discoverer Report from Poochon Scientific contained all assembled proteins with peptide sequences and peptide spectrum match counts (PSM#) and TMT-tag-based quantification ratios.

### Immunohistochemistry (IHC)

Formalin-fixed paraffin-embedded colonic biopsy sections from patients with intestinal BD (*n* = 20) and those with CD (*n* = 20) were stained with 39 antibodies and counterstained with hematoxylin–eosin. Antibodies against APOH (HPA003732) and MGAM (HPA002270) were purchased from Atlas Antibodies AB (Bromma, Sweden). Detailed antibody information, antibody dilution factors, and antigen retrieval methods are provided in Supplementary Table I. IHC analysis was performed as previously described^23^. Primary antibodies (1:1000 dilution for anti-APOH and 1:2000 dilution for anti-MGAM) were applied overnight at 4 °C. Staining was visualized using an Olympus BX43 microscope with the Olympus CellSens Entry software (Hamburg, Germany).

We used a semi-quantitative grading method to assess protein expression, as described previously^24^. Briefly, 100 × magnification was used to grade all fields for each sample, and the staining intensity was scored from 1 to 3. Staining extent was scored from 1 to 4. The scores were multiplied together, and the final scores were classified as follows: 1–3, weak; 4–8, moderate; and 9–12, strong staining. Fisher’s exact test was used to assess the immunochemical scores for protein expression. In this particular test, values of *p* less than 0.2 were determined as a threshold for moving to the next validation test.

### Enzyme-linked immunosorbent assay (ELISA)

Serum samples from patients with BD (*n* = 20) and those with CD (*n* = 20)—constituting an independent cohort of patients distinct from those providing samples for IHC staining—were analyzed using ELISA. The serum was diluted 1:50,000 for APOH and 1:200 for MGAM. ELISA was performed according to the manufacturer’s instructions using the APOH ELISA kit (KA0982, Abnova, Taipei, Taiwan) and the MGA ELISA kit (MBS2021345, MyBioSource, San Diego, CA).

### Data analysis and statistical methods

A heat map was generated using the “limma” package for R^25^. The fold changes of proteins and *p* values were calculated using linear regression in “limma.” The heat map was drawn using the heatmap.2 function in the “gplots” package for R (https://CRAN.R-project.org/package=gplots), and colors were changed using the “RColorBrewer” package for R (1.1-2. https://CRAN.R-project.org/package=RColorBrewer). Volcano plots were plotted using GraphPad Prism 8 software. Data were expressed as the mean ± SEM. Student’s *t-test* was used for the statistical analysis of serum ELISA data. *Statistical significance* was set at *p* < 0.05.

## Result

### Descriptive patient characteristics

The colon mucosa tissue samples of 27 patients with intestinal BD and 27 patients with CD, and serum samples of 20 patients with intestinal BD and 20 patients with CD, were obtained. Detailed patient characteristics of the 94 patients are shown in Table 1. The mean age of the patients with intestinal BD and CD was 51. 0 ± 11.4 years and 36. 1 ± 16.0 years (*p* < 0.001), respectively, which were comparable to those obtained in a previous study^7^. A total of 42.6% of the patients with intestinal BD and 74.5% of the patients with CD were men. The mean disease duration of intestinal BD and CD was 7.1 ± 6.0 years and 8.0 ± 5.5 years, respectively. Oral ulcers, genital ulcers, skin manifestations, and joint manifestations were more common in patients with intestinal BD and perianal lesions were more common in those with CD. Intestinal BD ulcers were located in the ileocecal valve (74.5%), ascending colon (10.6%), and postoperative anastomosis site (14.6%). The intestinal BD ulcers occurred with the following frequency: solitary (48.9%), two (19.1%), and multiple (31.9%). The CD showed localization in the ileal (14.9%), colonic (2.1%), and ileocolic (83.0%) regions and could be characterized as non-stricturing and non-penetrating (63.0%), stricturing (15.2%), and penetrating (21.7%). The number of patients with a DAIBD score ≥ 40 was 35 (74.5%) and that of patients with a CDAI score ≥ 150 was 24 (51.1%).

**Table 1:** Baseline characteristics of the study patients.

Variables	Intestinal Behƈet's disease (*n* = 47)	Crohn's disease (*n* = 47)	*p* value
Age (years)	51.0 ± 11.4	36.1 ± 16.0	< 0.001
Males	20 (42.6%)	35 (74.5%)	0.001
Smoking history	12 (25.5%)	11 (23.4%)	0.810
Disease duration (years)	7.1 ± 6.0	8.0 ± 5.5	0.439
Charlson comorbidity index (≥ 2)	8 (17.0%)	2 (4.3%)	0.045
**Extraintestinal manifestation**			
Oral ulcer	16 (41.0%)	0 (0.0%)	< 0.001
Genital ulcer	11 (28.2%)	0 (0.0%)	< 0.001
Skin manifestation	9 (23.1%)	0 (0.0%)	0.001
Joint manifestation	12 (30.8%)	2 (4.3%)	0.001
Eye manifestation	4 (10.3%)	3 (6.5%)	0.533
Vascular manifestation	1 (2.6%)	0 (0.0%)	0.459
Perianal lesion	1 (2.1%)	28 (59.6%)	< 0.001
**Disease activity score**			
Remission (CDAI < 150)	23 (48.9%)		
Mild (CDAI : 150–219)	10 (21.3%)		
Moderate (CDAI : 220–450)	13 (27.7%)		
Severe (CDAI > 450)	1 (2.1%)		
Remission (DAIBD < 20)		7 (14.9%)	
Mild (DAIBD : 20–39)		5 (10.6%)	
Moderate (DAIBD : 40–74)		26 (55.3%)	
Severe (DAIBD ≥ 75)		1 (2.1%)	
Previous surgery	10 (21.3%)	11 (23.4%)	0.804
**Previous medication**			
Corticosteroid	15 (31.9%)	15 (31.9%)	1.000
Immunomodulator	15 (31.9%)	21 (44.7%)	0.203
Anti-TNFɑ	4 (8.5%)	6 (12.8%)	0.503

Variables are expressed as mean ± SEM or *n* (%).

*BD* Behƈet's disease, *CD* Crohn's disease, *TNF* tumor necrosis factor, *CDAI* Crohn's disease activity index, *DAIBD* disease activity index of Behƈet's disease, *SD* standard deviation.

### Discovery of biomarker candidates using TMT/LC–MS/MS-based proteomic approach

**Figure 1:**
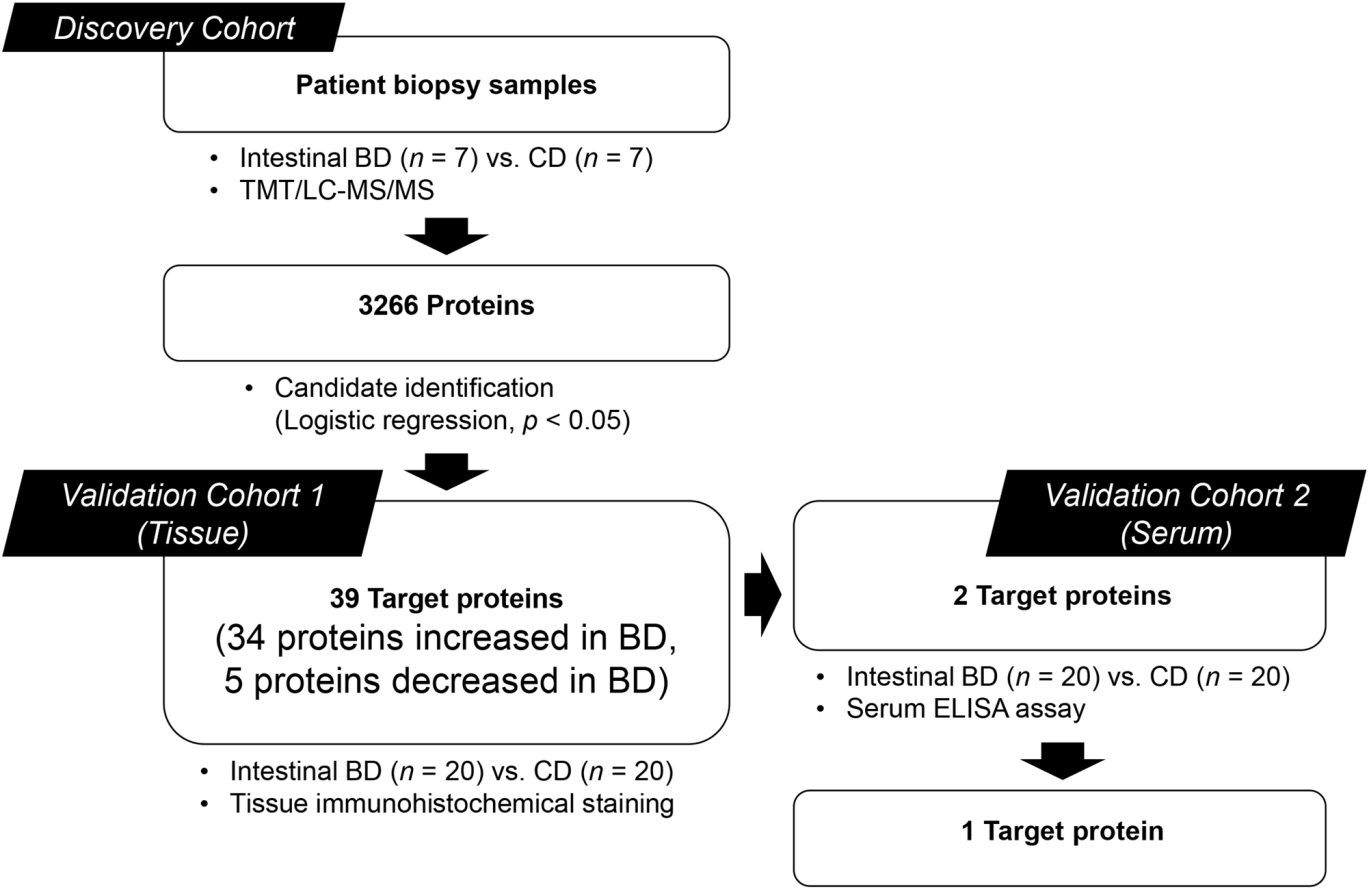
The colon mucosa tissue samples were obtained and analyzed using a TMT/LC–MS/MS-based approach for the discovery cohort (intestinal BD, *n* = 7; CD, *n* = 7). Candidate protein biomarker validation was performed using tissue IHC staining with the validation cohort 1 (intestinal BD, *n* = 20; CD, *n* = 20) and serum ELISA testing with the validation cohort 2 (intestinal BD, *n* = 20; CD, *n* = 20).

**Figure 2:**
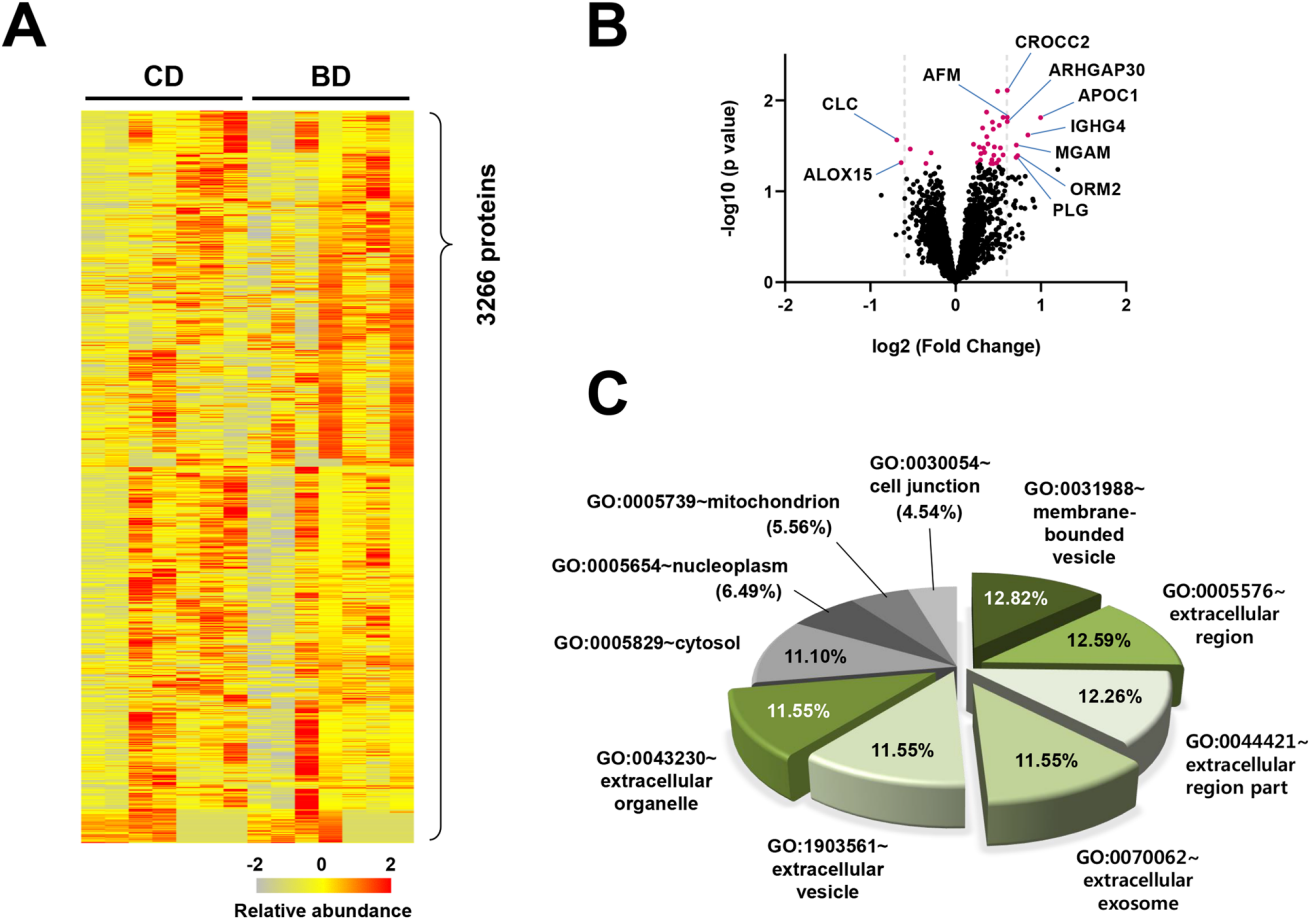
Proteomic characterization of 14 samples (seven from patients with intestinal BD and seven from patients with CD) using TMT-10plex labeling-based quantitative proteomics. (**A**) A heat map showing the relative abundance of 3266 proteins identified across two groups of human intestinal samples. The color key indicates the relative abundance of each protein (− 2 to 2) across 14 samples. (**B**) Volcano plot demonstrating fold changes in protein abundance between intestinal BD and CD. The *x*-axis represents the log2 ratio, and the *y*-axis represents significant differences (-log10 of *p* value). Proteins showing significantly altered expression (*p* < 0.05) are colored in magenta, and include the top ten up- or down-regulated proteins (fold change > 1.5). (**C**) A pie diagram showing cellular localization of human colon mucosa proteomes classified by the top ten cellular compartments in functional annotation with gene ontology (GO) with secretion-related proteins highlighted in green.

The colon mucosa tissue samples of patients with intestinal BD (*n* = 7) and those with CD (*n* = 7) were analyzed using a TMT/LC–MS/MS-based proteomic approach to identify intestinal BD-specific markers (Fig. 1). A total of 3,266 proteins were quantitatively identified, and at least two identified peptides were detected for each protein (Fig. 2A). Logistic regression analysis showed that 39 proteins were significantly different between intestinal BD and CD patients (*p* < 0.05) (Fig. 2B). Among them, 34 proteins were overexpressed, and 5 proteins were under-expressed in patients with intestinal BD compared to patients with CD (Table 2). The proteins were classified by cellular compartment (CC) based on gene ontology (GO) enrichment analysis using a functional annotation tool (DAVID Bioinformatics Resources, version 6.8) (Supplementary Table II). Among the top ten CC categories, 72.31% of GO items were associated with membrane-bounded vesicle (GO:0031988), extracellular region (GO:0005576), extracellular region (GO:0044421), extracellular exosome (GO:0070062), extracellular vesicle (GO:1903561), and extracellular organelle (GO:0043230), indicating that although there were a certain number of proteins from other cellular compartments such as the cytosol, nucleoplasm, mitochondrion, or cell junction, a large portion of colon mucosal proteomes were composed of secretion-related proteins (Fig. 2C). Notably, APOH and MGAM were included in all six secretion-related CC categories in the GO analysis.

**Table 2:** Candidate protein biomarkers for differential diagnosis between intestinal Behƈet's disease and Crohn's disease.

UniProt Entry name	Protein (full name)	UniProt Accession #	Gene name	Ratio (CD/BD)	*p* value	AUC
APOC1_HUMAN	Apolipoprotein C-I	P02654	APOC1	0.43	0.015	0.7755
IGHG4_HUMAN	Immunoglobulin heavy constant gamma 4	P01861	IGHG4	0.54	0.024	0.8163
PLMN_HUMAN	Plasminogen	P00747	PLG	0.61	0.042	0.8776
AFAM_HUMAN	Afamin	P43652	AFM	0.61	0.015	0.7347
RHG30_HUMAN	Rho GTPase-activating protein 30	Q7Z6I6	ARHGAP30	0.61	0.017	0.7347
A1AG2_HUMAN	Alpha-1-acid glycoprotein 2	P19652	ORM2	0.62	0.040	0.7959
MGA_HUMAN	Maltase-glucoamylase, intestinal	O43451	MGAM	0.63	0.031	0.7551
CRCC2_HUMAN	Putative ciliary rootlet coiled-coil protein 2	H7BZ55	CROCC2	0.66	0.008	0.8367
ZA2G_HUMAN	Zinc-alpha-2-glycoprotein	P25311	AZGP1	0.66	0.040	0.7755
ACE_HUMAN	Angiotensin-converting enzyme	P12821	ACE	0.67	0.015	0.8016
APOH_HUMAN	Beta-2-glycoprotein 1	P02749	APOH	0.68	0.033	0.8061
GILT_HUMAN	Gamma-interferon-inducible lysosomal thiol reductase	P13284	IFI30	0.69	0.019	0.7959
NHRF1_HUMAN	Na( +)/H( +) exchange regulatory cofactor NHE-RF1	O14745	SLC9A3R1	0.69	0.008	0.7959
VTDB_HUMAN	Vitamin D-binding protein	P02774	GC	0.70	0.045	0.7347
CLUS_HUMAN	Clusterin	P10909	CLU	0.70	0.049	0.7347
LOX5_HUMAN	Arachidonate 5-lipoxygenase	P09917	ALOX5	0.71	0.040	0.7959
LRRF1_HUMAN	Leucine-rich repeat flightless-interacting protein 1	Q32MZ4	LRRFIP1	0.71	0.033	0.7959
JUPI1_HUMAN	Jupiter microtubule associated homolog 1	Q9UK76	JPT1	0.72	0.049	0.8163
IL16_HUMAN	Pro-interleukin-16	Q14005	IL16	0.72	0.021	0.7755
C1S_HUMAN	Complement C1s subcomponent	P09871	C1S	0.73	0.017	0.8776
ASH2L_HUMAN	Set1/Ash2 histone methyltransferase complex subunit ASH2	Q9UBL3	ASH2L	0.76	0.049	0.4959
SRSF2_HUMAN	Serine/arginine-rich splicing factor 2	Q01130	SRSF2	0.76	0.030	0.8571
SRSF3_HUMAN	Serine/arginine-rich splicing factor 3	P84103	SRSF3	0.77	0.037	0.7959
ITA5_HUMAN	Integrin alpha-5	P08648	ITGA5	0.77	0.025	0.7755
PP4C_HUMAN	Serine/threonine-protein phosphatase 4 catalytic subunit	P60510	PPP4C	0.77	0.033	0.7347
AKP13_HUMAN	A-kinase anchor protein 13	Q12802	AKAP13	0.77	0.045	0.7551
TARA_HUMAN	TRIO and F-actin-binding protein	Q9H2D6	TRIOBP	0.77	0.013	0.8571
LMNB1_HUMAN	Lamin-B1	P20700	LMNB1	0.78	0.037	0.7755
TOIP2_HUMAN	Torsin-1A-interacting protein 2	Q8NFQ8	TOR1AIP2	0.80	0.020	0.9184
ACAP2_HUMAN	Arf-GAP with coiled-coil, ANK repeat and PH domain-containing protein 2	Q15057	ACAP2	0.82	0.045	0.8163
PLEC_HUMAN	Plectin	Q15149	PLEC	0.82	0.038	0.7755
SMRC2_HUMAN	SWI/SNF complex subunit SMARCC2	Q8TAQ2	SMARCC2	0.83	0.033	0.8571
LRRF2_HUMAN	Leucine-rich repeat flightless-interacting protein 2	Q9Y608	LRRFIP2	0.84	0.048	0.7857
NU205_HUMAN	Nuclear pore complex protein Nup205	Q92621	NUP205	0.87	0.030	0.8776
IMPA2_HUMAN	Inositol monophosphatase 2	O14732	IMPA2	1.22	0.038	0.7245
DMD_HUMAN	Dystrophin	P11532	DMD	1.26	0.049	0.7959
PERE_HUMAN	Eosinophil peroxidase	P11678	EPX	1.44	0.034	0.8163
LOX15_HUMAN	Arachidonate 15-lipoxygenase	P16050	ALOX15	1.53	0.048	0.7755
LEG10_HUMAN	Galectin-10	Q05315	CLC	1.59	0.027	0.8367

*BD* Behƈet's disease, *CD* Crohn's disease, *AUC* area under the curve.

### IHC validation of biomarker candidates

**Figure 3:**
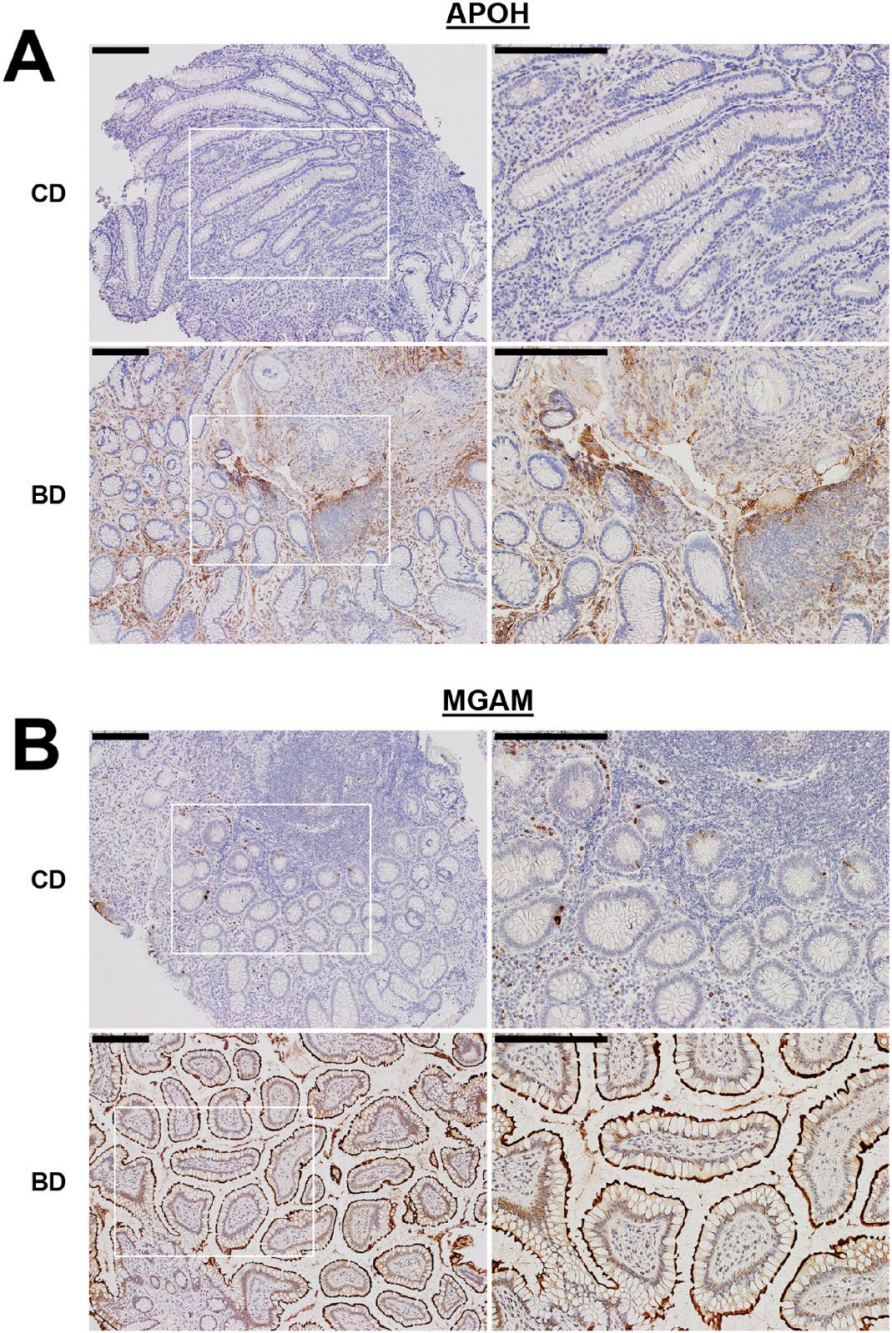
APOH and MGAM expression in the colonic mucosa. Tissue sections from patients with CD or BD stained with antibodies against APOH (**A**) or MGAM (**B**). The selected areas denoted by white boxes in left panels (original magnification, 100 ×) are enlarged in the right panels (200 ×). Scale bar, 200 µm.

IHC analysis was performed to validate 39 candidate biomarkers. A total of 40 colonic biopsy sections from patients with intestinal BD (*n* = 20) and CD (*n* = 20) were stained with each antibody and analyzed using a semi-quantitative grading method as described in the *Methods* section. Seven candidate proteins were selected based on their differential expression between intestinal BD and CD: maltase-glucoamylase (MGAM), beta-2 glycoprotein 1 (APOH), plasminogen (PLG), pro-interleukin 16 (IL16), serine/arginine-rich splicing factor 3 (SRSF3), clusterin (CLU), and serine/threonine-protein phosphatase 4 catalytic subunit (PPP4C). Fisher’s exact test showed that APOH (*p* = 0.039) and MGAM (*p* = 0.192) levels were consistently higher in intestinal BD than in CD, suggesting that these two proteins might be distinctive biomarkers of intestinal BD distinguishing it from CD. There were no significant differences in the expression of PLG (*p* = 0.480), IL16 (*p* = 0.563), SRSF3 (*p* = 0.591), CLU (*p* = 1.000), or PPP4C (*p* = 0.450) between intestinal BD and CD. IHC analysis of the colon mucosa tissue obtained from patients with intestinal BD showed that stronger APOH immunoreactivity was detected in a portion of the lamina propria (Fig. 3A, lower panels), and the MGAM immunoreactive signal was exclusively present in the brush border membrane of the epithelium (Fig. 3B, lower panels), consistent with previous findings^26^.

### Serum ELISA validation of biomarker candidates

**Figure 4:**
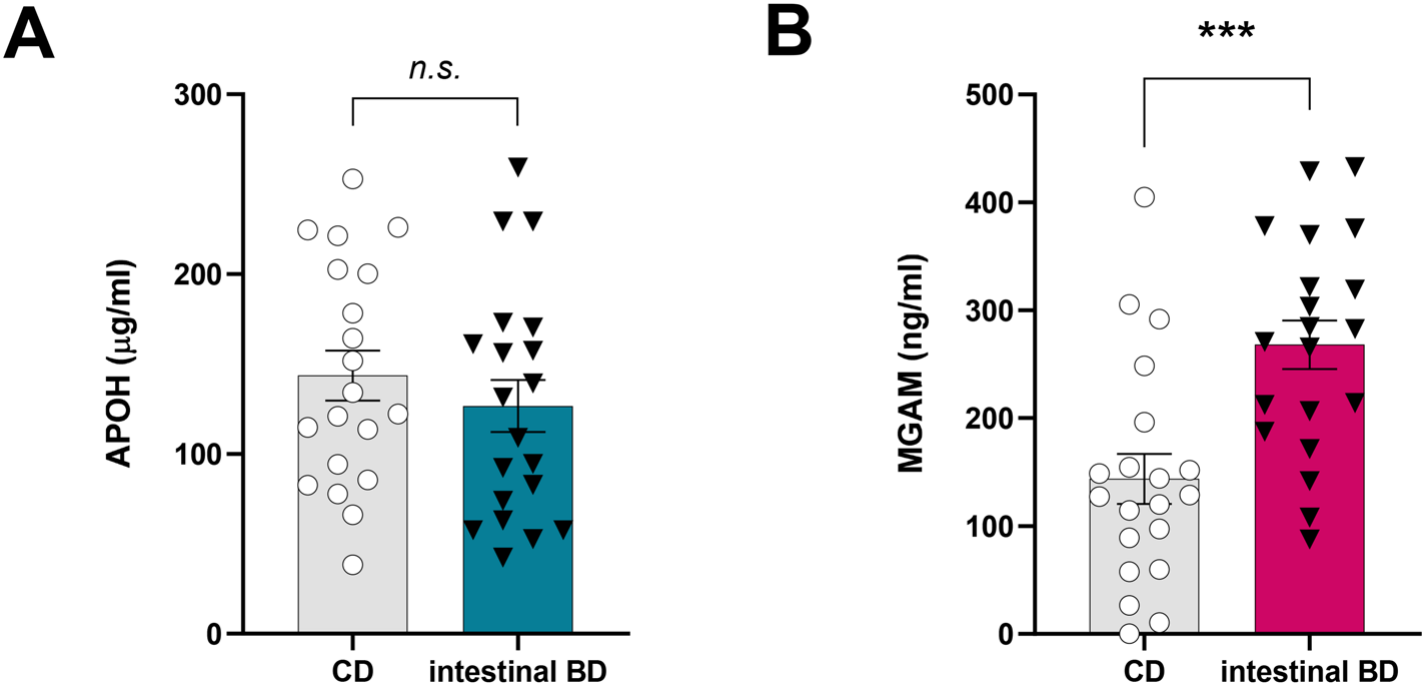
Serum APOH and MGAM protein expression detected using ELISA in intestinal BD patients (**A**) and CD patients (**B**). Results are mean ± SEM; ***p < 0.001. *n* = 20 patients per each group.

Since APOH and MGAM were included in all six secretion-related CC categories of GO analysis (Fig. 2C), we hypothesized that these proteins could also be detected in the blood. This notion was supported by several biochemical studies that identified APOH as a component of circulating plasma lipoproteins^27^, and the fact that MGAM is found in brush border membrane vesicles^28^. Both proteins were also recently detected in exosomes using in-depth proteomic analyses^29^. Although there was no difference in APOH concentration between the two groups (Fig. 4A), serum concentrations of MGAM were statistically higher in patients with intestinal BD compared to those with CD (*p* < 0.05), when the two candidate biomarkers were further tested with serum ELISA in an independent validation cohort (intestinal BD, *n* = 20; CD, *n* = 20) (Fig. 4B). These results suggest that MGAM can be a specific, diagnostic biomarker of intestinal BD.

### Analysis of the MGAM-related pathway in the colon mucosa proteome

**Figure 5:**
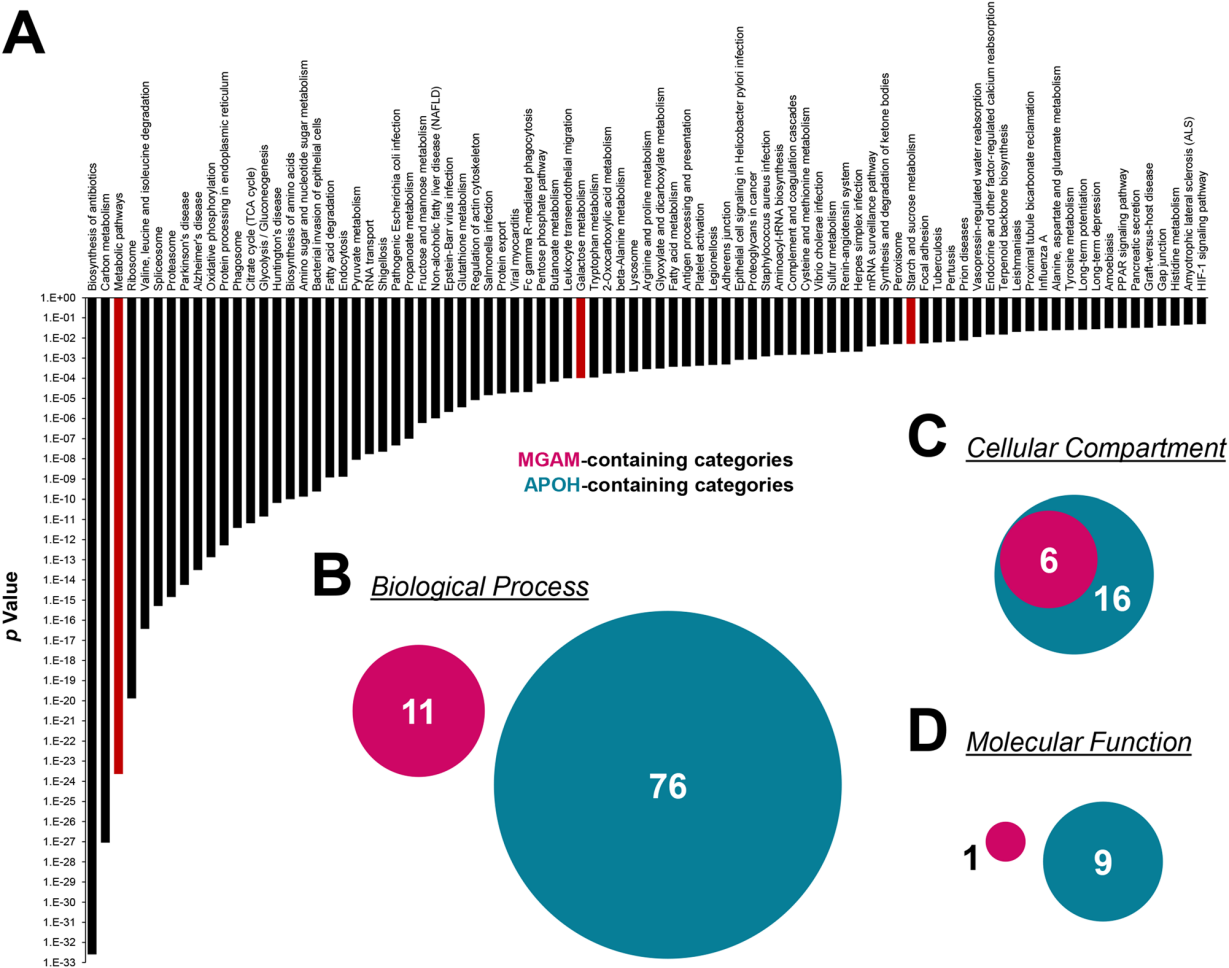
MGAM and APOH characterization in human colon mucosal proteomes. (**A**) Significant biological pathways enriched among 3,266 proteins identified by TMT are represented as bar graphs (*p* < 0.05) with the MGAM-containing categories highlighted in red. Venn diagrams showing numbers of MGAM-and APOH-containing categories classified by cellular compartment (**B**), molecular function (**C**), and biological process (**D**) based on functional annotation with gene ontology (GO) analysis.

The DAVID functional annotation tool was used to identify the biological pathways of the colon mucosa proteome of intestinal BD and CD patients, in which MGAM could be involved. The Kyoto Encyclopedia of Genes and Genomes (KEGG) demonstrated that 85 pathways were significantly enriched (*p* < 0.05) (Fig. 5A). Among them, MGAM was involved in three enriched pathways: metabolic pathways (hsa01100, 467 proteins), galactose metabolism (hsa00052, 19 proteins), and starch and sucrose metabolism (hsa00500, 17 proteins). Unexpectedly, APOH was not related to any of the 85 enriched pathways.

To determine whether MGAM and APOH share biological functions, 3,266 proteins were divided into three GO annotation categories: cellular compartment (CC), molecular function (MF), and biological process (BP). Next, the categories containing MGAM or APOH were selected and compared. There were six CC categories, but no MF or BP categories, correlating with both MGAM and APOH as represented in Figs. 2C and 5B–D. This suggests that the physiological roles of MGAM and APOH might differ in the colon mucosa, but both are assumed to be secreted.

### Predicted upstream regulators and disease-related functions of MGAM in intestinal BD and CD

**Figure 6:**
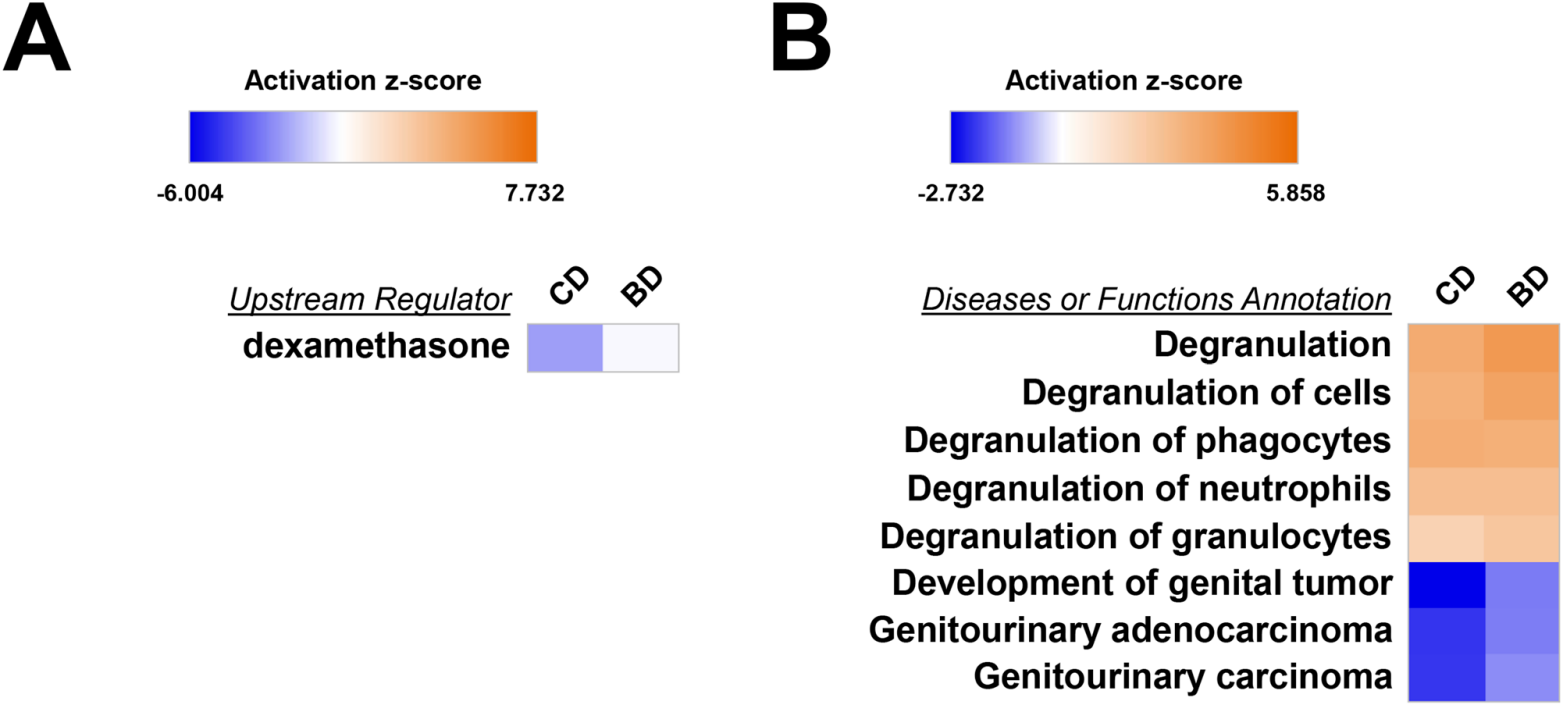
Ingenuity pathway analysis (IPA) of the MGAM-related upstream regulator (**A**) and disease or functional annotations (**B**) in colon mucosa proteomes of internal BD and CD. IPA core analyses were performed for a proteomic comparison between internal BD and CD using 3,614 UniProt accession numbers. Significant MGAM-related functional annotations are shown (z-score > 2, *p* < 0.05). Heat maps illustrating the predicted activation z-score. The color range indicates its predicted activation state: increasing (orange) or decreasing (blue).

Ingenuity pathway analysis (IPA) was performed to predict canonical pathways, upstream regulators, and disease-related functions to gain more insight into the pathophysiological role of MGAM in intestinal BD and CD. As shown in Supplementary Fig. 1A, the only canonical pathway related to MGAM was enriched in “Glycogen degradation III.” Although absolute z scores (CD, − 0.302; BD, 0.302) were lower than the cut-off (z-score > 2), the pathway significantly differed between intestinal BD and CD (*p* < 0.000001). The pathway was predicted to be inhibited in CD and activated in intestinal BD (Supplementary Fig. 1B). Upstream regulator analysis revealed that the dexamethasone-related pathway was inactivated in CD compared to its status in intestinal BD (Fig. 6A), but the medication history of corticosteroids (31.9%) was similar between the two patient groups participating in this study (Table 1). This result is consistent with previous observations that patients with CD require corticosteroid therapy more often than patients with intestinal BD^7^. Comparison of the diseases and functional analyses of the two patient groups revealed that the activation of degranulation and the inactivation of genitourinary carcinogenesis were conserved, and the former is predicted to be more upregulated in intestinal BD than in CD, and the latter is more downregulated in CD than in intestinal BD (Fig. 6B).

## Discussion

Accurate diagnosis of intestinal BD and CD is important for establishing proper treatment plans and predicting disease prognosis^30^. Clinical, laboratory, and endoscopic approaches have limitations in differentiating between these two diseases. Gastrointestinal and systemic symptoms, elevated inflammatory markers, and endoscopic findings, such as asymmetric deep ulcers in the ileocecal valve, are often shared between the two diseases in clinical practice^31^.

We quantified 3,266 proteins from the colon mucosa tissue samples, identified 39 novel diagnostic biomarkers, and validated the MGAM protein as a novel diagnostic biomarker using patient serum samples. To the best of our knowledge, this study is the first to use proteomics to identify a diagnostic marker that can differentiate intestinal BD from CD.

Quantitative proteomic analysis using isobaric chemical labeling, including super-stable isotope labeling with amino acids in cell culture, isobaric tags for relative and absolute quantitation, or TMT, is emerging as a highly effective approach with good quantification performance and reproducibility for profiling new biomarkers in numerous diseases^32^. These relatively new proteomic techniques enable the discovery of diagnostic biomarkers by providing methods not only for peptide identification, but also for the quantification of biological samples. Thus far, they have been applied to IBD^33^ or intestinal TB^24^, but not to intestinal BD. We identified 39 potential novel diagnostic biomarkers using quantitative proteomic analysis, and validated MGAM as a biomarker for differentiating intestinal BD from CD.

MGAM is involved in carbohydrate digestion in the small intestine. MGAM deficiency has been reported in congenital diarrheal diseases^34^. MGAM and sucrase-isomaltase (SI) have identical exon structures. They are anchored in the small intestinal mucosal brush border and hydrolyze substrates to glucose^35^. *Romach* et al. reported a trinitrobenzene sulfonic acid-induced colitis rat model that showed a loss of SI expression and activity^36^. *Lackeyram* et al. observed that a dextran sodium sulfate-induced colitis piglet model revealed decreased maximal specific activities of MGAM and SI^37^. Here, the tissue expression and serum concentrations of MGAM were lower in patients with CD patients than in those with intestinal BD. Our IPA analysis also predicted that the MGAM-related “glycogen degradation III” pathway was inactivated in CD (Supplementary Fig. 1A,B). Consistent with our findings, the involvement of the small intestine is relatively common in CD than in intestinal BD, resulting in digestive problems and nutrient malabsorption. Thus, differential MGAM expression may be related to different clinical manifestations between intestinal BD and CD, which should be further validated.

MGAM has been shown to be important in neutrophil biology but not in lymphocytes^38,39^. We identified MGAM in the colon mucosa tissue samples, but MGAM was also detected in the serum samples. The detected serum MGAM may be secreted from the gastrointestinal tract or contained in neutrophils. All five degranulation-related pathways (degranulation, degranulation of cells, degranulation of phagocytes, degranulation of neutrophils, and degranulation of granulocytes) enriched in the disease or functional annotations with high activation z-score indicated that MGAM might be involved in the degranulation process in which mast cell activation is essential. Although little is known regarding the degranulation in intestinal BD, degranulation signaling in IBD has been implicated in the regulation of inflammatory responses in the gastrointestinal tract, where the largest population of mast cells in the body resides^40–43^. Higher MGAM levels were detected in intestinal BD patients than in CD patients, and this was related to innate immunity pathogenesis. Further studies are required to understand how MGAM influences immune cell degranulation and the resulting gut inflammation. Moreover, it would be valuable to determine whether MGAM levels correlate with the disease activity of intestinal BD.

**Figure 7:**
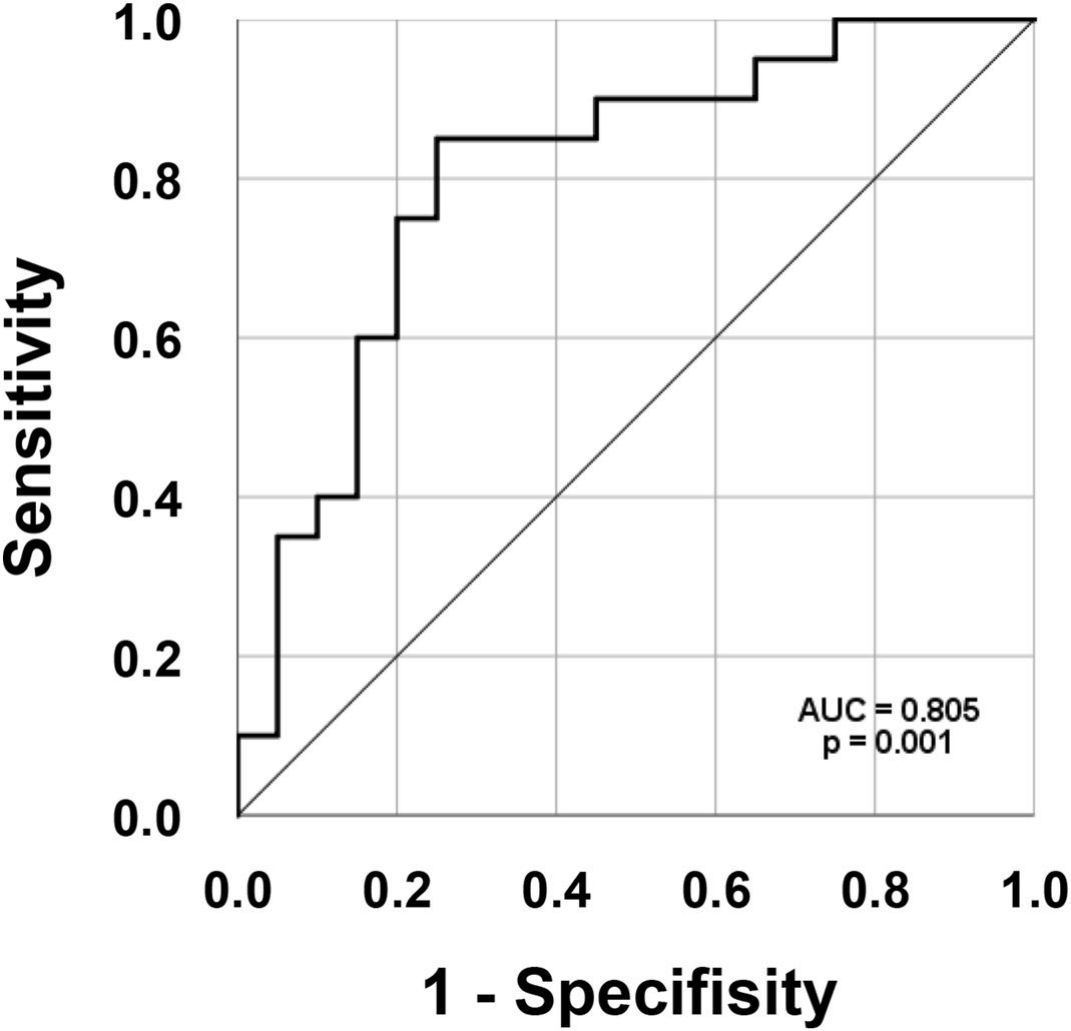
Receiver operating characteristic (ROC) curve showing the prediction of differentially diagnosing intestinal BD from CD using serum MGAM protein expression detected using ELISA.

Here, MGAM was shown to differentiate BD from CD with an area under the curve of 0.805 (95% confidence interval, 0.665–0.945), 85% sensitivity, and 70% specificity at a cut-off of 150 ng/mL (Fig. 7). However, further studies with a larger sample size are warranted to validate our data on the role of MGAM in chronic enterocolitis and to understand its molecular mechanism in depth. In addition, a significant proportion of our CD cohort population had perianal involvement, which distinguishes CD from intestinal BD. Perianal lesions in CD occur more frequently in Korea than in Western countries, but the ideal cohort population for the development of diagnostic biomarkers should present common or resembling clinical, endoscopic, radiologic, and histologic features of the two diseases. Future studies using such a cohort will be needed to validate MGAM as a distinguishing diagnostic biomarker. Finally, the measurement of MGAM levels in a variety of inflammatory diseases such as ulcerative colitis and intestinal tuberculosis could be worthwhile.

In summary, we used TMT-based proteomic quantification to identify 39 candidate proteins that were differentially expressed between intestinal BD and CD. Then, we selected APOH and MGAM proteins as possible biomarkers for intestinal BD based on the results of IHC staining and semi-quantitative grading. Finally, we suggest that the levels of MGAM protein in patient serum can potentially be used to differentially diagnose intestinal BD from CD.

## Supplementary Information

Supplementary Information 1.

Supplementary Information 2.

Supplementary Information 3.

## Abbreviations

BD: Behçet’s disease
CD: Crohn’s disease
IBD: Inflammatory bowel disease
TMT: Tandem mass tag
LC–MS: Liquid chromatography-tandem mass spectrometry
DAIBD: Disease Activity Index for Intestinal Behçet’s Disease
CDAI: Crohn’s Disease Activity Index
IHC: Immunohistochemistry
DAVID: Database for Annotation, Visualization and Integrated Discovery
MGAM: Maltase-glucoamylase
APOH: Beta-2 glycoprotein 1
PLG: Plasminogen
IL16: Interleukin 16
SRSF3: Serine/arginine-rich splicing factor 3
CLU: Clusterin
PPP4C: Serine/threonine-protein phosphatase 4 catalytic subunit
KEGG: Kyoto Encyclopedia of Genes and Genomes
IPA: Ingenuity Pathway Analysis
HLA: Human leukocyte antigen
MICA: MHC class I related gene A
CRP: C-reactive protein
ESR: Erythrocyte sedimentation rate
ASCA: Anti-Saccharomyces cerevisiae antibodies
AAEA: Anti-alpha-enolase antibodies
UC: Ulcerative colitis
TB: Tuberculosis
ACE: Angiotensin-converting enzyme
MT-2: Metallothionein-2
