# Supplementary material for: Proteomic analysis-based discovery of a novel biomarker that differentiates intestinal Behçet’s disease from Crohn’s disease
Source: Sci Rep. 2021 May 26;11:11019. doi: 10.1038/s41598-021-90250-2 (PMC8155054; doi:10.1038/s41598-021-90250-2)
Supplement: Supplementary file 1 — Supplementary Information 1. [file 41598_2021_90250_MOESM1_ESM.docx]

**Supplementary Information**

**Proteomic analysis-based discovery of a novel biomarker that differentiates intestinal Behçet’s disease from Crohn’s disease**

Jihye Park, MD, PhD^1,2†^, Daeun Jeong^3,4†^, Youn Wook Chung, PhD ^3,5†^, Seunghan Han^3,4^, Da Hye Kim^3^, Jongwook Yu, MD ^1,2^, Jae Hee Cheon, MD, PhD^1,2*^, and Ji-Hwan Ryu, PhD ^3,4*^

^1^Department of Internal Medicine, Yonsei University College of Medicine, ^2^Institute of Gastroenterology, Yonsei University College of Medicine, ^3^Severance Biomedical Science Institute, Yonsei University College of Medicine, ^4^Brain Korea 21 PLUS Project for Medical Science, Yonsei University College of Medicine, and ^5^Airway Mucus Institute, Yonsei University College of Medicine, Seoul 03722, Korea

Running title: Biomarker differentiating intestinal BD from CD

*Ji-Hwan Ryu and Jae Hee Cheon contributed equally to this work.

^†^Jihye Park, Daeun Jeong, and Youn Wook Chung contributed equally to this work.

*Correspondence: [yjh@yuhs.ac](mailto:yjh@yuhs.ac)

Ji-Hwan Ryu, PhD

Severance Biomedical Science Institute, Yonsei University College of Medicine, 50-1 Yonsei-ro, Seodaemun-gu, Seoul 03722, Korea

Tel.: +82-(0)2-2228-0757

Fax: +82-(0)2-2228-1998

*Correspondence: [Geniushee@yuhs.ac](mailto:Geniushee@yuhs.ac)

Jae Hee Cheon, MD, PhD

Department of Internal Medicine, Yonsei University College of Medicine, 50-1 Yonsei-ro, Seodaemun-gu, Seoul 03722, Korea

Tel.: +82-(0)2-2228-1990

Fax: +82-(0)2-393-6884

**
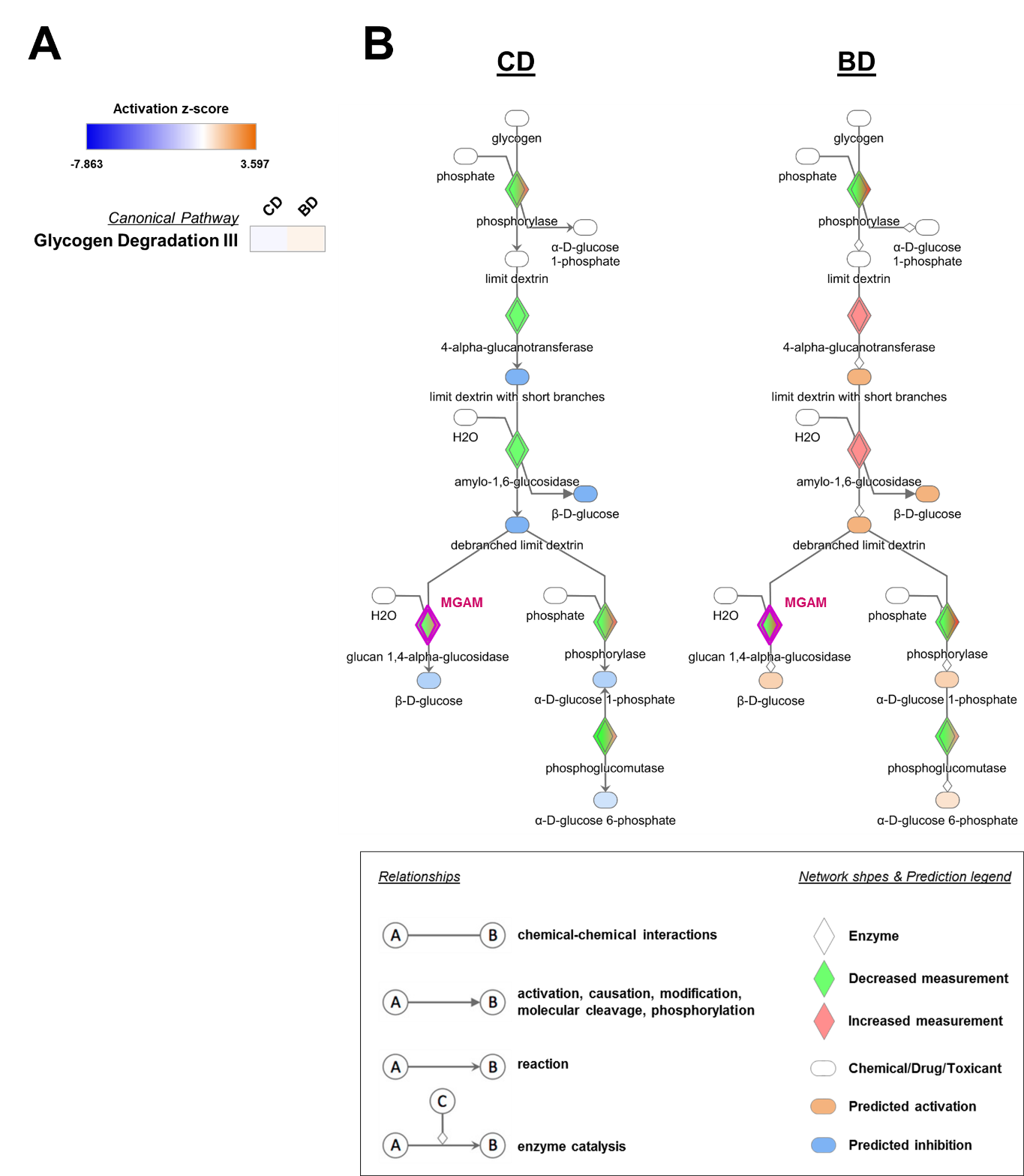
**

**Supplementary Figure. 1** Schematic of the MGAM-related canonical pathway, Glycogen Degradation III. MGAM has glucan 1,4-alpha-glucosidase activity.
